# Work-Related Stress among a Cohort of Italian Long-Term Care Workers during the COVID-19 Pandemic: An Observational Study

**DOI:** 10.3390/ijerph19105874

**Published:** 2022-05-12

**Authors:** Andrea Conti, Sophia Russotto, Annalisa Opizzi, Matteo Ratti, Daniele Nicolini, Kris Vanhaecht, Massimiliano Panella

**Affiliations:** 1 Department of Translational Medicine, Università del Piemonte Orientale, 28100 Novara, Italy; russottosophia@gmail.com (S.R.); 20022851@studenti.uniupo.it (A.O.); matteo.ratti@uniupo.it (M.R.); daniele.nicolini@uniupo.it (D.N.); 2 KU Leuven Institute for Healthcare Policy, 3000 Leuven, Belgium; kris.vanhaecht@kuleuven.be; 3 Aging Project Unit, Department of Translational Medicine, Università del Piemonte Orientale, 28100 Novara, Italy

**Keywords:** work-related stress, long-term care, healthcare workers mental health, COVID-19, mental health

## Abstract

Despite long-term care (LTC) workers having been identified as particularly subject to chronic stress, only a few studies evaluated the impact of the COVID-19 pandemic on stress in this population. As far as the authors know, no studies have investigated the relationship between work-related stress and chronic stress in the LTC setting. This retrospective observational study aimed to assess the level of chronic stress in LTC workers, to identify some possible predictors and vulnerability factors, and to measure the impact of the COVID-19 pandemic on work-related stress. The study was based on the information gathered from two different questionnaires administered before and one year after the beginning of the pandemic, to a cohort of Italian LTC workers. We found that chronic stress was associated with lower resilience to stress scores (57.42 vs. 60.66) and with higher work-related stress scores (30.48 vs. 20.83). Interestingly, the overall level of work-related stress did not differ between the two questionnaires (27.84 vs. 29.08). However, the main components of the questionnaires changed; fatigue and burnout symptoms became more relevant after the pandemic. Results of this study suggests deepening knowledge of the components of stress to develop and implement effective stress mitigation interventions.

## 1. Introduction

Psychological stress happens to an individual when the environmental requests exceed their adaptive capacity [1]. If the external trigger factor continues to exert its action for a long time, it may result in a chronic stress state. This condition plays a role in the development of a meaningful number of other chronic conditions, such as major depressive disorder or cardiovascular disease [2,3]. Among all the factors that are recognized to be chronic stressors, a central role is represented by the job of the individual. High workloads, night shifts, and staff shortages expose long-term care (LTC) workers to the risk of suffering from chronic stress [4,5]. In fact, their resilience is often limited, and many authors demonstrated the effectiveness of interventions, which promotes coping skills [6]. In addition, it was estimated that work-related stress carries a considerable cost for societies (ranging from 221 million to 187 billion of US dollars) and represents a major cause of productivity loss [7]. The recent pandemic provided an example of a sudden stressor which may interact with other chronic ones already experienced by LTC workers. Moreover, the length of the pandemic may have turned acute stressors into chronic ones.

Work-related stress is one of the major determinants of sick leave and productivity loss due to chronic stress disease [8,9]. Accordingly, LTC workers are significantly affected by chronic stress, having higher rates of burnout compared to the general population [10]. This suggests the presence of a vulnerable substrate on which sudden stressors can have a detrimental impact, generally due to heavy workloads, inadequate staffing, night shifts, and the grief caused by the deaths of patients [11,12].

During the COVID-19 pandemic, health services faced an incredible surge in demands. LTC had to deal with additional burdens due to a severe shortage of personnel, more shifts, longer shifts, a lack of personal protective equipment, and the low perceived safety conditions [13]. Current evidence shows that LTC patients are one of the most vulnerable populations to COVID-19 [14], as they have high mortality and fatality rates [15]. Additionally, during the COVID-19 pandemic, healthcare professionals experienced higher levels of stress, burnout, secondary trauma, anxiety, and depression, especially the frontline workers [16]. Although several studies assessed the psychosocial impact of the COVID-19 pandemic on healthcare workers, most of them were performed in hospital and in emergency settings; only a few evaluated the impact of the COVID-19 pandemic on LTC. For example, the presence of anxiety, post-traumatic symptomatology, stress, and insomnia among workers were assessed; but work-related stress and its relationship with chronic stress was not specifically addressed [17,18].

Therefore, we conducted a study aimed to assess the level of chronic stress in a cohort of LTC workers, and to identify some possible stress predictors and vulnerability factors. As a secondary goal, we evaluated the impact of the COVID-19 pandemic on LTC workers in terms of work-related stress.

## 2. Material and Methods

### 2.1. Study Design, Setting, and Participants

We performed an observational retrospective study using available information from a stress campaign promoted by the Italian not-for-profit company Anteo Impresa Sociale ONLUS. Anteo manages LTC services across Italy, having more than 3100 available beds among 200 services. Its geographical distribution of managed services and huge number of employees makes Anteo representative of the Italian LTC workers population. Anteo provides various initiatives to improve the well-being of its employees, such as lifestyle interventions and prevention campaigns [19]. In January 2020, a stress prevention and treatment campaign was started within corporate welfare initiatives. The campaign included a baseline questionnaire (Q1) designed to assess chronic stress, work-related stress, and resilience toward stress. According to the final score, people who took part in the campaign were invited to different counseling and prevention initiatives. The questionnaire was proposed on a voluntary basis to all the 1184 employees from 1 February 2020 to 29 February 2020 through the internal mailing list. Initially, the time to completion was planned to be two months; however, the beginning of the outbreak was chosen as the end of the initiative.

**Figure 1:**
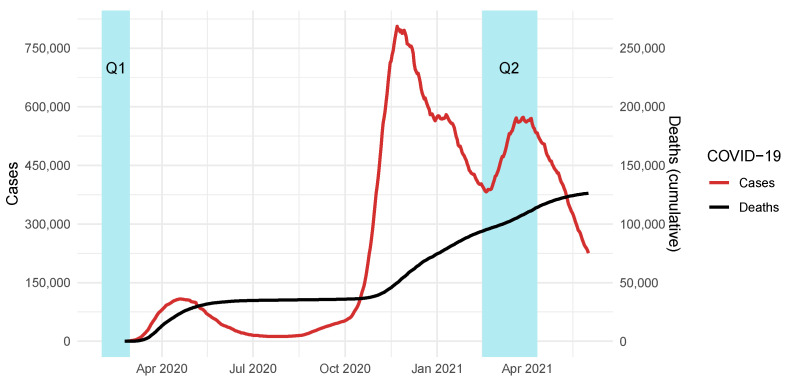
COVID-19 cases and death in Italy compared to questionnaires’ administration timing (Q1 and Q2).

In January 2021, Anteo welfare department decided to conduct a new survey to evaluate the impact of the COVID-19 pandemic on its workers. This initiative was designed to specifically screen the work-related stress of the LTC workers, with the aim of providing a rational basis with which to better address treatment and prevention initiatives. This second questionnaire (Q2) was administered from 15 February 2021 to 11 April 2021, just after the second wave of cases and deaths (Figure 1).

As the questionnaires were offered to all employees, different professionals were represented. Responders were subsequently considered “healthcare workers” if their jobs had direct and continuous contact with patients (e.g., nurses, health assistants, psychologists, and physiotherapists) or “clerks” if the contact with patients was absent or sporadic (e.g., administrative personnel, cooks, maintainers, and laundry employees).

### 2.2. Questionnaires

Both questionnaires were electronically built using SoGoSurvey software (2291 Wood Oak Drive, Herndon, VA, USA). Supplementary Table S1 provides the overview.

The Q1 questionnaire investigated chronic stress, work-related stress, and resilience to stress. In detail, chronic stress was investigated using the Kessler Psychological Distress Scale (KPDS) [20] (Supplementary Table S3), and work-related stress and resilience to stress were assessed through an adapted version of the Maslach Burnout Inventory (MBI) [21] and an adapted version of the Resilience Scale for Adults (RSA) [22] (Supplementary Tables S4 and S5). The survey contained, in total, 35 questions (10, 15, and 10 from KPDS, MBI, and RSA, respectively); the overall scores could range from 35 to 245. Low scores represent low levels of chronic stress, work-related stress, and resilience. In addition, specific thresholds were defined for each section. Values equal or higher than 13, 40, and 58 were identified as high levels of chronic stress, work-related stress, and resilience to stress, respectively, [23,24].

Differently, the Q2 questionnaire was specifically designed to assess work-related stress. It contained the fifteen questions from the MBI already implemented in Q1; the overall scores could range from 15 to 105. The threshold of 40 differentiated low versus high work-related stress.

The MBI and RSA Italian translations were obtained from previous studies [25,26]. In contrast, KPDS was translated from English to Italian by two different employees; then, the two versions were compared by a third individual to choose the best one. This process was deemed necessary, as no Italian translations of KPDS were found in the literature. Answers were collected through 7-level Likert scales (Supplementary Table S2).

### 2.3. Statistical Analysis

We used R 4.1.2 (R Core Team, Vienna, Austria) for data processing and statistical analysis.

Descriptive statistics described the population characteristics, namely, demographic and occupational factors. The Cronbach test was used to investigate the reliability of the questionnaires. The Shapiro–Wilk test was used to assess whether numerical variables were normally distributed. The significance level was set to 5% (α = 0.05); the calculated sample size for detecting a 25% effect size with a power of 80% was 100 respondents.

We conducted a univariate analysis of the respondents’ characteristics in Q1, to investigate whether individuals with and without chronic stress presented different demographic and occupational factors. We used the Mann–Whitney U test to assess the quantitative variables, and we used Fisher’s exact test for dichotomous variables.

As secondary analyses, we performed multiple simple linear regression and a principal component analysis (PCA) among the workers that answered both questionnaires (paired samples). More specifically, the multiple linear regression aimed to assess whether demographic characteristics, occupational factors, chronic stress, and resilience in Q1 were associated with the work-related stress measured in Q2. The PCA was performed for both questionnaires on the work-related stress section and aimed to explore the contribution of each answer to the overall score.

## 3. Results

A total of 505 responses were included in the analysis, 197 and 308 from Q1 and Q2, respectively. All Q1 respondents also completed Q2. Response rates were 17% and 26% for Q1 and Q2, respectively.

The Cronbach test showed good reliability for both questionnaires (α = 0.89 and α = 0.93, respectively). None of the quantitative variables were normally distributed. Demographic and occupational data are shown in Table 1; the two groups did not differ in age, seniority, gender, or contact with patients. However, most of the respondents were female, and around half of the respondents were healthcare workers.

Mean work-related stress and resilience were significantly higher and lower in the respondents with chronic stress, respectively. Among the respondents, healthcare workers showed a significantly greater level of chronic stress when compared to clerks (Table 2).

In the paired samples, work-related stress in Q2 was significantly associated with chronic stress in Q1 (Table 3).

**Figure 2:**
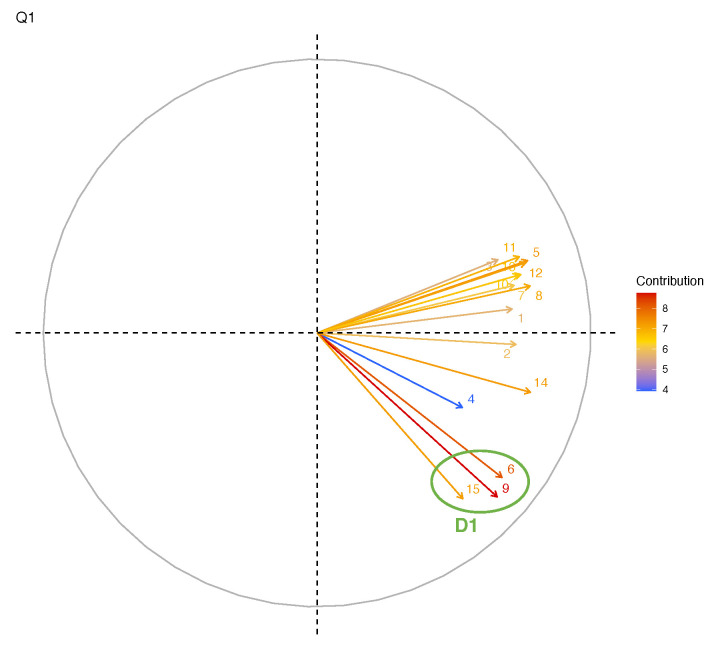

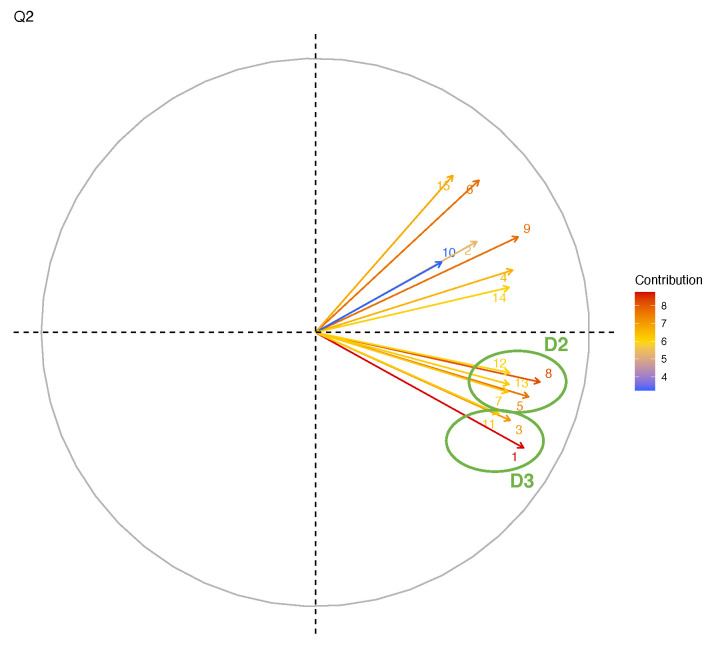
Principal component analysis of the work-related stress sections of the questionnaires. Each arrow represents a different question. The green circles highlight the identified domains.

Figure 2 shows the main components of work-related stress in Q1 and Q2, measured for the 197 respondents of both questionnaires. In detail, we identified in Q1 a main domain (D1) which included three questions on trust toward the personal commitments of their jobs. Differently, in Q2 we observed two different domains encompassing questions on work fatigue (D2) and burnout-related symptoms (D3). Table 4 shows the questions included in the domains. Specifically, questions 9 and 6, and questions 1 and 8 represented the main contributions to Q1 and Q2, respectively.

## 4. Discussion

As a major finding, our study showed that the presence of chronic stress at baseline was higher in healthcare professionals, in workers with low levels of resilience to stress, and in subjects affected by work-related stress. In contrast, we did not find any significant association with age, gender, or job seniority. These results confirmed previous studies in which healthcare workers were identified as a high-risk group for burnout, regardless of gender and age [10,16].

Not surprisingly, the work-related stress in Q2 was associated with chronic stress in Q1. None of the other factors, including resilience to stress in Q1, were significantly associated.

This was probably due to the fact Q2 was administered after the first COVID-19 surge, of which the devastating effects on mental health were described as having the potential to lead to post-traumatic stress disorder [27]. In such conditions, the behaviors, feelings, perceptions, and stress of people involved in disasters frequently change over time [28], and resilience to stress scores in Q1 could not be representative at the time of Q2. This could suggest a possible *harvesting effect* of the pandemic on pre-existing coping and adaptation mechanisms that could not be sufficiently strong to face sudden events. In fact, because of the pandemic, LTC workers have been directly exposed to harsh, unpredictable work environments, such as prolonged working hours, increased demands, and exposure to human suffering, for instance, through the increased number of deaths [29]. As a result, some are likely suffering from mental exhaustion due to excessive workload and the stress of not being able to take care of both patients and their families. Indeed, despite the overall work-related stress scores not differing between Q1 and Q2, the PCA showed significant differences in the main components of the work-related stress. Q2 work-related stress was characterized by a high level of fatigue and the presence of burnout symptoms, which can be reasonably attributed to the consequence of working during the pandemic [30,31]. In fact, despite non-frontline healthcare professionals such as LTC workers reporting milder symptoms than those working on the front line, they were equally damaged, suggesting the pandemic had a general detrimental impact on the well-being of all health sectors’ workers [32,33]. In contrast, Q1 work-related stress was mainly caused by low personal commitments of the assigned job. According to previous studies, this is a well-known phenomenon, generally attributed to a shortage of staff, high demands, and a poor environment [10,34,35]. In fact, despite symptoms such as anxiety and fear being important in the early stage of the pandemic, they were quickly overcome by persistent post-traumatic and depression symptoms [36]. Additionally, after the first COVID-19 surge, personal commitment to the assigned job could have been fostered by the *heroic phase*, in which celebrations of healthcare workers by the community raised the optimism and provided a sense of relief [28]. However, the well-known impact of the pandemic on healthcare workers’ mental health might lead to an exacerbation of other stress domains, such as the above-mentioned fatigue and burnout symptoms. This aspect is supported by a previous study [37] that showed how levels of stress-related symptoms tended to remain stable during the first year of pandemic in the general population.

Even though LTC represents an important sector of healthcare [38] (for example, in Italy, 1.1 million workers are employed in LTC, and 19 billion euros are spent on it every year [39]), LTC facilities are often affected by a lack of personnel [34,40]. Thus, it is fundamental to deeply understand the mechanism of post-pandemic stress among LTC workers, to support the personnel operating in this context. However, further research is needed.

Our study had some limitations. First, the analysis was based on current data, originally collected for a different purpose. In fact, we did not have any control over the information gathering, nor the diffusion of the questionnaire. In addition, response rates were low, particularly for Q1. Different reasons could have affected the participation. First, the attendance was voluntary and some employees could not be interested in the subsequent stress mitigation initiatives. Second, as COVID-19 added a burden to the work, employees could have been totally absorbed by the problems caused by the pandemic, overshadowing the initiative [41]. Third, no reminders were sent after the initial invitation, although they have been identified as an effective strategy to increase the response rate in web surveys [42]. All of these points could have affected the participation and could have led to selection bias, with respondents not being representative of the overall population. Furthermore, resilience to stress and chronic stress were measured only at baseline and could not be representative of the population at the time of Q2. However, our study suggests that the COVID-19 pandemic had an impact on the work-related stress among our sample of LTC workers. Indeed, fatigue and burnout-related symptoms emerged as the new main components of the work-related stress at the time of Q2. Evidence from the literature showed how work-related stress can have detrimental effects on different aspects of workers’ health, increasing the risks of psychiatric, neurologic, cardiovascular, and metabolic diseases [43,44,45,46]. Although some initiatives to reduce work-related stress were already evaluated [47], the results of this study suggest that a deep understanding of the components is fundamental to developing and implementing effective work-related stress mitigation interventions.

## 5. Conclusions

The recent COVID-19 pandemic made the health sector face an incredible surge in demands, leading to additional burdens among LTC workers. Nevertheless, the literature on chronic and work-related stress in this population is limited. Our study showed that, though the work-related stress scores did not change after one year of pandemic, the main determinants varied. Indeed, if at baseline they were mainly characterized by trust toward the personal commitments of the assigned job, after the first wave of COVID-19 cases, the domains of work fatigue and burnout-related symptoms were prominent. Results of this study suggest that is fundamental to foster a deep understanding of work-related stress components, with the aim of developing and implementing effective stress mitigation initiatives.

## Figures and Tables

**Table 1:** Demographic, occupational, and survey score information of the respondents.

	Q1 (N = 197)	Q2 (N = 308)	*p*-Value
Gender (female, N, rate)	159 (81%)	241 (78%)	0.57
Age (years, mean, CI95%)	44.33 (42.89–45.78)	45.38 (44.24–46.53)	0.17
Job seniority (years, mean, CI95%)	7.66 (6.89–8.43)	7.05 (6.46–7.64)	0.19
Healthcare worker (N, rate)	96 (49%)	143 (46%)	0.76
Chronic stress (score, mean, CI95%)	15.82 (15.01–16.64)	Not measured	-
Work-related stress (score, mean, CI95%)	27.84 (25.88–29.81)	29.08 (27.41–30.75)	0.65
Resilience (score, mean, CI95%)	58.86 (57.86–59.87)	Not measured	-

**Table 2:** Descriptive statistics of the 197 respondents to Q1. The tests compare respondents with a chronic stress scores higher and lower than the predefined threshold (13/70).

	Chronic Stress − (N = 86)	Chronic Stress + (N = 111)	*p*-Value
Age (years, mean)	45.40	43.32	0.21
Gender (female, n, mean)	67 (86%)	92 (83%)	0.47
Job seniority (years, mean)	7.62	7.7	0.83
Healthcare worker (N, rate)	69 (80%)	71 (64%)	0.020
Resilience (score, mean)	60.66	57.42	<0.001
Resilience (high vs. low, N, rate)	63 (73%)	53 (48%)	<0.001
Work-related stress (score, mean)	20.83	30.48	<0.001
Work-related stress (high vs. medium/low, N, rate)	0 (0%)	20 (18%)	<0.001

**Table 3:** Results of the multiple linear regression analysis of Q1 results. The dependent variable was the Q2 work-related stress score.

	Estimate	Std. Error	T Value	*p*-Value
Intercept	17.66	15.34	1.15	0.25
Healthcare worker	−1.13	2.43	−0.47	0.64
Gender (male)	−2.36	2.69	−0.88	0.38
Chronic stress score	1.20	0.25	4.81	<0.001
Resilience score	−0.01	0.22	−0.050	0.96
Age (years)	−0.14	0.12	−1.14	0.26
Seniority (years)	0.05	0.21	0.24	0.81

**Table 4:** Questions included in the identified domains.

Question Number (Domain)	Content
6 (D1)	I feel I do not give a constructive contribution to the organization.
9 (D1)	I feel I am not doing well my job.
15 (D1)	I have no faith in my professional skills.
5 (D2)	I feel burned out from of my job.
7 (D2)	I feel a big gap between my ambition and my job.
8 (D2)	I feel less enthusiast of my job than ever before.
12 (D2)	I feel frustrated by my job.
13 (D2)	I feel fatigued when I get up in the morning and have to face another day on the job.
1 (D3)	I feel mentally exhausted by my job.
3 (D3)	An entire working days is a heavy burden for me.
11 (D3)	I feel drained at the end of a working day.

## Data Availability

Data not available due to ethical and privacy restrictions.

## Supplementary Materials

The following are available at https://www.mdpi.com/article/10.3390/ijerph19105874/s1, Table S1: Questionnaires’ overview. Table S2: Likert scale values. Table S3: Kessler Psychological Distress Scale. Table S4: Maslach Burnout Inventory (adapted). Table S5: Resilience Scale for Adults (adapted).

## Abbreviations

The following abbreviations are used in this manuscript:

COVID-19	Coronavirus Disease 2019
LTC	Long-Term Care
Q1	Questionnaire 1
Q2	Questionnaire 2
PCA	Principal Component Analysis
KPDS	Kessler Psychological Distress Scale
MBI	Maslach Burnout Inventory
RSA	Resilience Scale for Adults
