# Supplementary material for: Work-Related Stress among a Cohort of Italian Long-Term Care Workers during the COVID-19 Pandemic: An Observational Study
Source: Int J Environ Res Public Health. 2022 May 12;19(10):5874. doi: 10.3390/ijerph19105874 (PMC9140776; doi:10.3390/ijerph19105874)
Supplement: Supplementary file 1 [file ijerph-19-05874-s001.zip › ijerph-1657246-supplementary.pdf]

Supplementary material

Table S1: Questionnaires' overview

Topic	Base	Present in Q1	Present in Q2	Questions
Chronic stress	Kessler Psychological Distress Scale (original version)	Yes	No	10
Work-related stress	Maslach Burnout Inventory (adapted version)	Yes	Yes	15
Resilience to stress	Resilience Scale for Adults (adapted version)	Yes	No	10

Table S2: Likert scale values

1	2	3	4	5	6	7
Never	Up to two times per year	Up to one time per month	Two times per month	Weekly	Two times per week	Daily

Table S3: Kessler Psychological Distress Scale

Question	Content
1	I feel tired for no good reason
2	I feel nervous without no good reason
3	I feel so nervous that nothing could calm me down
4	I feel hopeless
5	I feel restless or fidgety
6	I feel so restless I could not sit still
7	I feel depressed
8	I feel so sad that nothing could cheer me up
9	I feel that everything is an effort
10	I feel worthless

Reference scores: 10-12 no chronic stress; 13-70 chronic stress.

Table S4: Maslach Burnout Inventory (adapted)

Question	Content
1	I feel mentally exhausted by my job
2	I am doubtful about the usefulness of my job
3	An entire working day is a heavy burden for me
4	I have not accomplished many worthwhile things in this job
5	I feel burned out from my job
6	I feel I do not give a constructive contribution to the job
7	I feel a big gap between my ambition and my job
8	I feel less enthusiast of my job than ever before
9	I feel I am not doing well my job
10	I do not feel satisfied after completing my job
11	I feel drained at the end of a working day
12	I feel frustrated by my job
13	I feel fatigued when I get up in the morning
14	I have become more callous toward my job
15	I have no faith in my professional skills

Reference scores: 15-39 no work-related stress; 40-105: work-related stress.

Table S5: Resilience Scale for Adults (adapted)

Question	Content
1	I usually manage one way or another
2	I feel proud that I have accomplished things in my life
3	I do not dwell on things that I can not do anything about
4	I am friend with myself
5	I am determined
6	I keep interested in things
7	My belief in myself gets me through hard times
8	My life has meaning
9	When I am in a difficult situation, I can usually find my way out of it
10	I have enough energy to do what I have to do

Reference scores: 10-57 no resilience; 58-70: resilience.
